# List of Cases Treated in the Surgical Wards of the Pennsylvania Hospital

**Published:** 1839-01-26

**Authors:** T. Harris

**Affiliations:** Attending Surgeon


### CLINICAL REPORTS.

### PENNSYLVANIA HOSPITAL.

*List of Cases treated in the Surgical Wards of the Pennsylvania Hospital.* Dr. T. Harris, Attending Surgeon.

[Reported by H. H. Smith, M. D., Resident Surgeon.]

*Case of Strangulated Femoral Hernia,—Operation seventy-two hours after Strangulation,—Cured in twenty-six days.*

Eliza C—, coloured, aged forty years, was admitted on December 10th, with a strangulated femoral hernia. She stated, that she had been subject to hernia for the last eighteen months, and had always reduced it; that, at present, the bowel had been down seventy-two hours, that attempts had been made, by a physician in the city, but without success, and that the necessary means not being accessible, she was sent to the hospital. On her entrance, her pulse was feeble and quick—countenance expressive of suffering, great tenderness of the abdomen on pressure, with stercoraceous vomiting. After the use of the warm bath, and an injection, the operation for femoral hernia was performed by Dr. Harris. On opening the sac, more than a half pint of serum escaped from the abdomen. The intestine was much discoloured and inflamed, and the seat of stricture at Hey's ligament. The usual dressings were then applied, the wound having been brought together by the interrupted suture, and stimulants carefully given to the patient. Notwithstanding the severe peritonitis, and discharge of serum, which continued for some days, the wound healed kindly, and in twenty days after the operation she was permitted to walk, a soft padded truss having been previously applied.

